# Controlled fermentation and preservation of UGBA –an indigenous Nigerian fermented food

**DOI:** 10.1186/2193-1801-2-470

**Published:** 2013-09-17

**Authors:** Chimezie Princewill Okorie, Nurudeen Ayoade Olasupo

**Affiliations:** Federal Institute of Industrial Research Oshodi, Lagos, Nigeria; Department of Microbiology, Faculty of Science, Lagos State University Ojo, Lagos, Nigeria

**Keywords:** Starter culture, Controlled, Fermentation, Preservation

## Abstract

Studies were carried out to screen various microbial isolates of UGBA obtained from both traditionally fermented and laboratory samples for some technical properties required for the fermentation of the product. The technical properties screened for were; ability to produce enzymes (amylase, protease and lipase) and bacteriocin production. Possible starter cultures were selected from the screened isolates for controlled fermentation of the product. Preservation of the product by dehydration method was also investigated. Various dehydrating temperatures were studied and the most appropriate temperature regime was adopted. The shelf- life of the dehydrated product was also determined. Proximate composition and the amino acid profile of both fresh samples and the dehydrated ones were also carried out so as to ensure that there is no significant nutrient lost during the process of dehydration. Rehydration of the preserved product was also examined. The following groups of organisms were isolated; *Bacillus* species, *Proteus* species, *Staphylococcus* species, *Micrococcus* species and *Pseudomonas* species. *Bacillus* species exhibited the highest potential for the fermentation of the product based on the result of the technical properties screened for. Two isolates identified as *Bacillus subtilis* and *Bacillus lichenformis* were particularly outstanding and were therefore selected as possible starter cultures. Controlled fermentation of UGBA using the selected organisms singly and as mixed culture produced samples that were similar to the ones produced by the traditional method. However, fermentation period was reduced from 72 hr to 48 hr using the two isolates as mixed culture for the fermentation process. The most appropriate temperature regime for dehydrating the product was found to be 50°C. Proximate analysis and amino acid profile assay of the products show that there is no significant difference between the preserved product and fresh sample. Shelf- life studies of the samples showed that there is a significant difference between the preserved sample and the fresh one in terms of their keeping quality (6 months and 3 days respectively). The fresh sample lost its integrity (colour, taste, texture and aroma) after 72 hours while the preserved sample maintained its integrity even after six months of storage under room temperature.

## Introduction

‘UGBA’ refers to fermented African oil bean (*Pentaclethra macrophylla Benth*) seeds which are utilized by the Ibos and other ethnic groups in southern Nigeria as a delicacy and food flavoring (Ikenebomeh *et al*., 1986). The oil bean seed is mainly composed of proteins (42%), lipids (43%) and carbohydrates (15%) (Odunfa and Oyeyiola, 1985; Isichei *et al*., 1988, Njoku and Okemadu, 1989; Ogueke and Aririatu, 2004).

Published studies on the microbiology of the fermentation of African oil bean seeds have identified *Bacillus* spp as the main microorganisms responsible for its fermentation. The predominant species is *Bacillus subtilis* but other species like *B. pumilus, B. megaterium, B. lichenformis* have also been found (Odunfa, 1981; Antai and Ibrahim 1986; Odunfa and Oyewole, 1986; Diawara et al. 1998). The same group of organisms has been implicated in the fermentation of other fermented food condiments like Iru, Dawadawa, Soumbala, Afiyo and Ogiri.

The traditional fermentation methods widely practiced in Africa and other developing countries usually involve a spontaneous development of different lactic acid producing bacteria. The final microbiological status of the products so derived by these methods is influenced in part by the raw materials and the process method (Steinkraus, 1983). These process methods have almost always led to the problem of inconsistent product quality and other attendant problems. In Africa, majority of the fermented foods are produced at household level and hygiene is a major concern (Olasupo *et al*., 2002, Gadaga *et al*., 2008).

The problem of occurrence and growth of pathogens in most of these fermented food products cannot be ruled out as the general hygienic conditions of the processors, the equipment used, water and other raw materials cannot be said to be free of potential pathogens.

The use of starter cultures has generally been recognized as one major way of ensuring product consistency and to a reasonable extent eliminates the problem of food-borne pathogens (Eman, 2009). Unfortunately however, no lactic acid bacteria (LAB) starter cultures are commercially available yet for small scale processing of traditional African foods. The potential of starter cultures for fermentation on a household scale for most of our traditionally fermented foods has not yet been fully explored.

A starter culture is applied to improve a fermentation process, be it a lactic, alcoholic or the other types of fermentation. The old tradition of using a portion of a fermented product to start a new batch resembles the principle of starter culture in an empirical sense. However, most commercial starter cultures originated from those food substrates to which they are applied today.

Another major problem encountered by the local processors of this product is the poor keeping quality. The product has a shelf-life of about three (3) days. This implies that the processor has to dispose off his product within three days of production beyond which time the product goes bad and is thrown away.

The aim of this work therefore was to screen various microbial isolates of ugba for technical properties required for the fermentation of the product and subsequently select starter culture(s) for its production and to examine possible ways of preserving it so as to extend the shelf life. The achievement of these objectives will help to reduce the problem of occurrence of pathogens and improve the poor keeping quality of the product being experienced by the local processors.

## Results and discussion

Studies on isolation and identification of the fermenting microorganisms of African oil bean seeds identified the following groups of microorganisms as being present during the fermentation of African oil bean seed into UGBA; *Bacillus* species, *Proteus* species, *Micrococcus* species, *Staphylococcus* species and *Pseudomonas* species (Table 1). This study was designed to identify the organisms responsible for the fermentation of the African oil bean seeds into UGBA and ultimately select starter cultures for its production. The presence of members of the coliform group which were earlier isolated and considered as possible pathogens in this study were therefore disregarded as they are unlikely to be selected as possible starter cultures for the production process.

**Table 1:** **Microscopic, biochemical and physiological properties of extracellular enzymes producing organisms isolated from fermenting ugba**

Characterization test	Bacterial isolate				
	1	2	3	4	5
Gram reaction	+	-	+	+	-
Catalase	+	+	+	-	-
Casein Hydrolysis	+	+	+	-	+
Gelatin Liquefaction	+	+	-	-	+
Starch Hydrolysis	+	+	+	-	-
Voge-Proskaeuer	+	-	+	+	+
Citrate Utilization	+	+	-	+	-
Oxidase	ND	ND	ND	ND	ND
H_2_S Production	+	-	-	-	+
Urease	+	+	-	+	+
**Sugar fermentation**					
Fructose	A	A	A	A	-
Galactose	A	-	A	-	-
Glucose	A	-	AG	A	A
Lactose	A	A	A	-	-
Maltose	A	A	A	-	A
Mannitol	A	-	A	A	A
Sucrose	A	A	A	A	-
Xylose	ND	ND	ND	ND	ND
Tentative identity	Bacillus	Proteus	Staphylococcus	Micrococcus	Pseudomonas

KEY: + = Positive, - = Negative, *ND* = Not done.

*Bacillus* species have been implicated in the fermentation of most vegetable oil protein seeds like African locust bean seeds, soy bean seeds, African oil bean seeds etc (Odunfa, 1981; Antai and Ibrahim 1986; Odunfa and Oyewole, 1986; Diawara et al. 1998; Ogueke and Aririatu, 2004 ). Some workers in their study of microbial and organoleptic changes associated with UGBA stored at ambient temperature, identified *Proteus* species as one of the predominant species isolated throughout the period of their study (Ogueke and Aririatu, 2004). *Staphylococcus* species and *Micrococcus* species have also been isolated from fermenting African oil bean seeds.

*Pseudomonas* species have rarely been reported to be associated with fermentation of UGBA or any other vegetable protein seeds. Oral reports however suggest that in some instances, this organism could be involved in the fermentation process of UGBA as cases of products occasionally having a shade of greenish colour have been cited by the local processors. Some species of *Pseudomonas* are noted for a greenish –gray- bluish colour production. It is therefore possible that such shade of greenish colour occasionally observed by the local processors could have been as a result of the presence of *Pseudomonas* species in such products. It is however possible that they are pre-fermentation contaminants as they were only detected at the early stages of the fermentation process in this study. However, further work needs to be conducted to determine if they could play any active role in the fermentation process of UGBA. This based on the fact that they were found to possess the ability to produce protease enzyme that has been established to play key role in the fermentation of the product.

The traditional method of fermentation of most African fermented foods is by chance inoculation, which implies that one of the factors that determine the organisms to be isolated from such fermenting product will be the initial microbial content of the starting raw materials (water inclusive). *Pseudomonas* species are reported to be a natural inhabitant of water bodies. It is therefore possible that the occurrence of this species of organism in this work could have originated from the source of water used in the processing of the product.

### Screening of isolates for enzyme production

**Figure 1:**
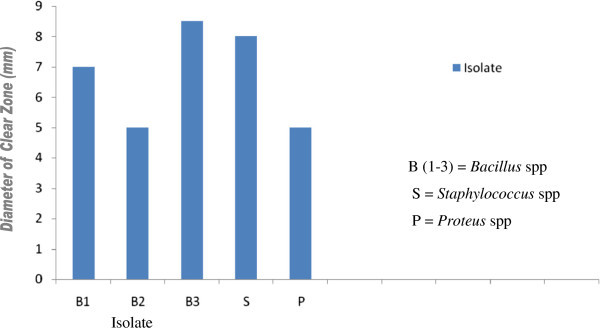
**Production of amylase by micro-organisms isolated from fermenting African oil bean seeds.**
- Protease:
  Investigation conducted earlier on isolation and identification of organisms responsible for the fermentation of African oil bean seeds into ugba shows that among the major groups of organisms responsible for the fermentation of Ugba are the *Bacillus* species, *Proteus* species, *Staphylococcus* species, *Micrococcus* species and *Pseudomonas* species. Recorded reports however show that the major group of organisms associated with the fermentation of ugba is the *Bacillus* species. Therefore the *Bacillus* species isolated in this work were subjected to further identification process to determine their strain. Result obtained shows that twelve (12) different strains of *Bacillus* species identified as *Bacillus subtilis* ( Bs1, Bs2, Bs3, Bs4, Bs5, Bs6 and Bs7) and *Bacillus lichenformis* ( B_l_1, B_l_2, B_l_3 and B_l_4) were among the *Bacillus* species isolated ( Table 2).
  These different strains of *Bacillus* species*, Proteus, Staphylococcus, Micrococcus and Pseudomonas species* were screened for protease enzyme production since the major activity during Ugba production has been established to be proteolytic in nature. The result of this study shows that all the species of *Bacillus* isolated were able to produce protease enzyme. Also, *Proteus* and *Pseudomonas* species were able to produce protease enzyme (Figure 2). Isolate Bs3 recorded the highest production rate while isolates Bl2 and Bl3 had the least production rate as expressed by the diameter of clear zone observed for each isolate.

- Amylase:
  The screening of various isolates obtained from fermenting African oil bean seeds for their ability to produce amylase enzyme revealed that the *Bacillus* species, *Staphylococcus* species and *Proteus* species have the capacity to produce amylase (Figure 1). Most of the previous workers on the production of Ugba (Odunfa, 1981; Antai and Ibrahim 1986; Odunfa and Oyewole, 1986; Diawara et al. 1998; Ogueke and Aririatu, 2004 ) have mainly associated the *Bacillus* species with the production of protease and by extension with the proteolytic activities associated with the production of the product. Other workers have also associated the Bacillus species with amylolytic activity (Oguntimei, 1993). The result of this work has however shown that the *Bacillus* species which is one of the predominant organisms during the production of UGBA are not only capable of producing protease enzyme which play a key role in the fermentation process but are also active in the production of amylases. *Bacillus* species has however been identified as important sources of proteases and amylases (Forgarty *et al.*^1974^).

**Figure 2:**
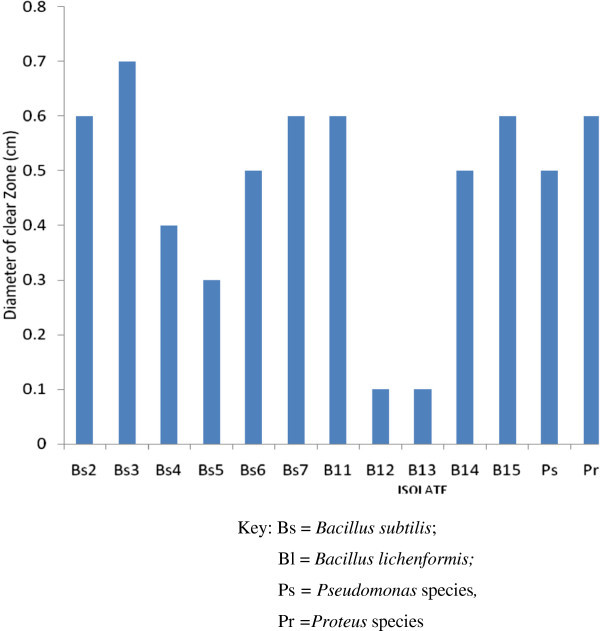
**Protease enzyme production by microorganisms isolated from ugba.** Key: Bs = *Bacillus subtilis*; Bl = *Bacillus lichenformis;* Ps *= Pseudomonas* species*,* Pr *= Proteus* species Bacterial Isolate.
  The result obtained in this work identifies *Bacillus* species (*B. subtilis* and *B*. *lichenformis*) as one of the major groups of organisms responsible for the fermentation of ugba. Similar results have been recorded by various workers in the past (Odunfa, 1981; Antai and Ibrahim, 1986; Odunfa and Oyewole, 1986;Diawara et al. 1998; Ogueke and Aririatu, 2004). These workers observed that the fermentation of ugba and other related vegetable proteins is usually by alkaline fermentation brought about by the activities of *Bacillus* species. Similar observations have been recorded with the fermentation of Iru, Dawadawa, Soumbala, Afiyo and Ogiri where the same group of organisms have been implicated in their production (Obeta, 1983; Odunfa and Oyeyiola 1985;Njoku et al., 1990; Diawara et al. 1998; Ouoba et al. 2003. Omafuvbe, *et al.*^2004^; Ogunshe, *et al*^2007^.).
  Reports available show that the major activity taking place during fermentation of Ugba is proteolysis (Odunfa and Oyeyiola, 1985; Njoku and Okemadu, 1989 and Ogueke and Aririatu 2004). They associated the *Bacillus* species with this proteolytic activity. The result of this work which identifies all the *Bacillus* species isolated from fermenting UGBA as being able to produce protease enzyme is therefore in agreement with their findings.
- Lipase:
  The screening of isolates for lipase production showed that *Micrococcus* species, *Pseudomonas* species and a strain of *Bacillus subtilis* (Bs2*)* were able to produce lipase enzyme. These isolates have been selected based on the diameter of clear zone expressed on the culture plate by them and will be subjected to further studies.

**Table 2:** **Microscopic, biochemical and physiological properties of** ***Bacillus*** **species isolated from fermenting African oil bean seed**

Bacterial strains												
Xterization tests	Bs1	Bs2	Bs3	Bs4	Bs5	Bs6	Bs7	B11	B12	B13	B14	B15
**Gram reaction**	+	+	+	+	+	+	+	+	+	+	+	+
**Catalase**	+	+	+	+	+	+	+	+	+	+	+	+
**Casein hydrolysis**	+	+	+	+	+	+	+	+	+	+	+	+
**Gelatin liquefaction**	+	+	+	+	+	+	+	+	+	+	+	+
**Starch hydrolysis**	+	+	+	+	+	+	+	+	+	+	+	+
**Methyl red**	+	-	+	+	-	+	+	+	+	+	+	-
**Voges-Proskaeuer**	+	+	+	+	+	-	+	+	+	+	+	+
**Citrate utilization**	+	+	+	+	+	+	+	+	+	+	+	+
**Oxidase**	ND	ND	ND	ND	ND	ND	ND	ND	ND	ND	ND	ND
**H**_**2**_ **S production**	+	+	+	+	+	+	+	+	+	+	+	+
**Sugar fermentation**												
**Fructose**	A	A	A	A	A	A	A	A	A	A	A	A
**Galactose**	A	A	A	A	A	A	A	A	A	A	A	A
**Glucose**	A	A	A	A	A	A	A	AG	A	AG	AG	A
**Lactose**	A	A	A	A	A	A	A	A	A	A	A	A
**Maltose**	A	A	A	A	A	A	A	A	A	A	A	A
**Mannitol**	A	A	A	A	A	A	A	A	A	A	A	A
**Sucrose**	A	A	AG	A	A	A	A	AG	A	A	A	A
**Xylose**	ND	ND	ND	ND	ND	ND	ND	ND	ND	ND	ND	ND

Key: + = positive, - = negative, *A* = acid production, *AG* = acid production with gas, *ND* = Not determined, *Bs* = *Bacillus subtilis*, *Bl* = *Bacillus licheniformis*.

## Controlled fermentation of ugba

A preliminary work on controlled fermentation of African oil bean seeds into ugba using the tentatively selected starter cultures show that all the *Bacillus* isolates were able to ferment the seeds into ‘ugba’ going by the texture, colour taste and aroma of what was obtained at the end of the fermentation process. Isolates B_3_ and B_4_ identified as *Bacillus subtilis* and *Bacillus lichenformis* were outstanding in terms of the quality of ugba they produced. These two isolates were therefore selected for further work on the controlled fermentation of ugba. Further work revealed that there was no significant difference in both physical and chemical properties of products obtained by starter culture fermentation and the traditional fermentation (Table 3).

**Table 3:** **Physical and chemical properties of starter culture fermented and traditionally fermented ugba**

Property	Starter culture	Traditionally fermented
	fermented ugba	ugba
Taste	characteristic of ugba	characteristic of ubga
Color	Brownish	Brownish
Texture	Soft	Soft
pH	7.8	7.6
Time{hrs}	48	72
Sliminess	Slightly slimy	Slightly slimy

There was however a significant reduction in fermentation period (48 hrs) when a mix culture of isolates B_3_ and B_4_ were used for a controlled fermentation of ugba as compared with the spontaneously fermented sample where fermentation was achieved in 72 hrs (Table 4).

**Table 4:** **Proximate analysis of starter culture fermented and traditionally fermented ugba**

Composition	Traditionally	Fermented	Fermented	Fermented by
	fermented	by isolates	by isolates	isolates B3
	sample	B3	B4	and B4
Fat	10.51	15.50	12.09	15.03
Ash	1.09	1.68	0.98	1.21
Calcium	0.98	0.80	1.20	0.98
Magnesium	0.75	0.60	1.00	0.90
Protein (undefatted)	13.17	14.96	13.85	14.76
Moisture	49.77	42.01	47.10	43.78
Crude Fiber	17.64	16.51	16.36	16.66
Carbohydrate	7.82	9.34	9.66	9.12

## Proximate analysis of samples

Proximate compositional analysis of samples produced by both the traditional method of fermentation and controlled fermentation method showed that there is no significant difference between them (Table 5). However, there was a significant difference in the protein content recorded in this work and what has been reported earlier by Pierson *et al.* (1986), Sanni, (1993), Eka, (1980). While they reported a 43.9% protein content, 14.96% was recorded in this work. It is however believed that the 43.9% they reported in their work was for defatted sample, while the 14.96% recorded in this was for undefatted sample.

**Table 5:** **Amino acid profile analysis of traditionally fermented, starter culture fermented and dehydrated ugba.**

Amino acids traditionally fermented	Starter culture	Preserved/dehydrated	
	sample(% )	fermented sample (%)	sample (%)
Arginine	1.94	1.98	1.95
Cystine	0.64	0.88	0.76
Methionine	0.26	0.40	0.32
Histidine	1.03	1.21	1.20
Isoleucine	1.25	1.15	1.13
Leucine	2.39	2.35	2.34
Lysine	2.11	2.15	2.15
Phenylalanine	1.34	1.45	1.40
Threonine	0.96	1.03	1.00
Tryptophan	0.31	0.26	0.22
Valine	1.29	1.46	1.44
Alanine	0.78	0.88	0.67
Glycine	0.86	0.79	0.79
Glutamic acid	2.95	2.87	2.86
Aspartic acid	1.37	1.54	1.47

### Amino acid assay

There was no significant difference in the amino acid composition of both the traditionally fermented samples and the starter culture fermented ones. Preservation of the product by dehydration method was also studied and there was also no observed difference in the amino acid composition of the dehydrated sample and the other two samples analyzed (Table 6).

**Table 6:** **Changes in some physical and chemical properties of dehydrate Ugba with increase in storage time**

Time{wk}	pH	Moisture content	Color	Aroma	Texture
0	7.55	3.20	Normal	Normal	Brittle
4	7.56	3.20	Normal	Normal	Brittle
8	7.53	3.25	Normal	Normal	Brittle
12	7.54	3.23	Normal	Normal	Brittle
16	7.52	3.26	Normal	Normal	Brittle
20	6.93	4.56	Normal	Normal	Brittle
24	6.46	5.86	Normal	Normal	Soften
28	5.55	14.67	Normal	Slightly Off	Soften
32	4.48	16.77	Slightly off	Off	Soften
			color (darker )		
s36	4.46	30.45	Off	Off	Soften
					& sticky

### Preservation and shelf-life studies

Preservation studies on the product were done by dehydration method. Various dehydrating temperature regimes were studied, but dehydrating temperature of 50°C was found most appropriate in terms of colour and other physical properties monitored.

Shelf-life studies conducted show that the dehydrated samples maintained their integrity even 6 months after production, showing a remarkable improvement in the keeping quality of the product which does not normally last beyond 3 days (Table 6).

 (There is no reported record on the preservation of ugba using dehydrated method. However, attempts have been made in the past to extend the shelf-life of this product through chemical and other preservative methods. An attempt was made in the past to package the product in low and high density polyethylene sachets and aluminum foil as well as treatment with chemical preservatives such as 2% sodium chloride Ogbulie *et al.*^1998^). However, none of the methods could extend the shelf-life beyond 8 days. Some workers in their study applied the process of pasteurization at a temperature of 98-100°C for 30 min, which they said completely eliminated all the organisms present including the organisms used for the fermentation (Mbata and Orji 2008). This was only able to extend the shelf-life for 8 days. They also made attempts to package their product in returnable and sterilizable bottles/cups. These were only able to keep the product for six weeks. The result recorded in this work is therefore a significant improvement on what have been reported in literature and could go a long way in solving the problem of poor keeping quality of the product being experienced by the local processors.

## Conclusion

This study has shown the *Bacillus* species as the major fermenting organisms for the production of ugba and could therefore be selected as starter cultures for a controlled fermentation of the product. Preservation of the product by dehydrating it below 10% moisture content level significantly improved the keeping quality of the product. This result will go a long way towards solving the problem of poor keeping quality of the product being reported by the local processors.

## Materials and methods

### Laboratory preparation of Ugba

The traditional method of preparing Ugba was employed in the laboratory to ferment the product (Pierson *et al.,*^1986^). The processing of the large brown glossy seeds of the African oil bean to obtain ‘Ugba’ involved the following; the oil bean seeds were boiled in an autoclave at a temperature of 121°C and a pressure of 15 pounds per square inch (psi) for 1 hr to soften the hard brown testa (shell). The shells were removed and the kernels washed, drained and rewashed with cold water several times. The washed cotyledons were cut into long thin slices. These slices were wrapped in small packets with leaves and lightly tied. These small packets were placed in a basket to ferment at room temperature for 3 days to yield ‘Ugba’.

### Microbial analysis

Samples were collected daily from the fermenting African oil bean seeds in the laboratory and randomly from the following local markets within Lagos metropolis; Ajegunle, Oshodi, Cele along Oshodi Mile 2 expressway Okokomaiko and Umuahia in Abia state Nigeria. Serial dilutions of the samples were prepared and the dilutions were plated out on nutrient agar and tryptone soy agar using the spread plate method. Based on the cultural and morphological characteristics of the organisms, different isolates were selected and purified by streaking on the same media as used in the isolation process. Characterization and identification of the isolates were done based on the cultural and morphological features of the isolates on the plates, sugar fermentation and biochemical tests carried out and the use of API 50 CHB Kit. Further biochemical tests and sugar fermentation tests were carried out to differentiate the *Bacillus* isolate into strains.

### Screening of isolates for enzyme production

- Amylase:
  The plate assay technique was employed in the screening of the isolates for amylase production. Nutrient agar medium with 1% inclusion of soluble starch was prepared; the isolates were inoculated on each plate and incubated for 48 hr at 37°C (Ramakrishna et al. (1982). At the end of the incubation period, the plates were flooded with iodine solution and observed for colour change around the growth of each isolate. A change of colour from the usual blue – black colour of iodine on starch to light yellow indicated a positive result, while a blue – black colour indicated a negative result.
- Protease:
  The plate assay technique was employed in screening the isolates for protease enzyme production. Nutrient agar with 1% inclusion of casein was prepared; the isolates were smeared on the plates of the medium and incubated at 37°C for 48 hrs. The plates were observed for clear zone at the end of the incubation period. Clear zone around each isolate indicated a positive result while no clear zone signified a negative result.
- Lipase:
  The same organisms isolated from fermenting African oil bean seeds were screened for the production of lipase enzyme. The plate assay technique was also employed in the screening exercise. These organisms were inoculated on a tributytrin agar medium and incubated at 37°C for 72 hr. These organisms were observed for a clear/hallo zone around each isolates on the plate daily throughout the incubation period. A clear/hallo zone around the isolates indicated a positive result, while no clear zone indicated a negative result.

The quantitative assay of these enzymes has been reported by Okorie and Olasupo (2013).

### Controlled fermentation of ugba

Preliminary investigation on a controlled fermentation of ugba using isolates selected as possible starter cultures was carried out. The isolates were grown in nutrient broth for 72 hr in a shaker incubator at 150 rpm. The cells were harvested by centrifugation at 5000 rpm. The cells were re-suspended in sterile distilled water and centrifuged again to wash off the nutrient broth, this was done three times and the cells suspended in sterile water. The cells of each isolate were seeded into sterilized sliced African oil bean seeds and left to ferment for 72 hr. At the end of the fermentation period, the fermenting seeds were assessed for taste, colour,smell and texture to determine the level of fermentation.

## Proximate analysis of samples

The proximate analysis of the starter culture produced ‘Ugba’ and the traditionally fermented ones were carried out. The following parameters were determined; protein, fat, carbohydrate, ash, crude fiber, moisture, calcium and magnesium contents. These analyses were done according to standard methods. The experiments were done in triplicates.

## Spectrophotometric determination of amino acids using ninhydrin chemical reaction method

Determination of the amino acid content of the samples was done using the Ninhydrin chemical reaction method (Schroeder *et al.*^1950^, Spies and Chambers, 1950). The experiments were done in triplicates*.* Ninhydrin combines with amino acid to form colored complexes, the intensity of those colors depend on the amount of amino acid present.

## Preservation by dehydration and shelf-life studies

Freshly prepared ugba samples were dehydrated in a vacuum pump oven. The following dehydrating temperature regimes were examined; 45°C, 50°C, 55°C, 60°C, 65°C and 70°C. 50°C dehydrating temperature was selected based on the quality of product obtained in terms of texture, color and aroma of product. Subsequent dehydration was therefore done at 50°C. Dehydration process lasted for about 4 hrs and the final moisture content of dehydrated product determined. Moisture content was calculated by drying samples at 130°C to a constant weight (AOAC 1975).

Dehydrated samples were packaged in polyethylene bags, sealed and left on the shelf for 9 months to determine the shelf-life. Samples were taken from these and examined for changes in color, pH, moisture content, texture and taste at a weekly interval throughout the 9 months period.
